# Measurement of kynurenine pathway metabolites by tandem mass spectrometry

**DOI:** 10.1016/j.jmsacl.2023.04.003

**Published:** 2023-04-15

**Authors:** Sedat Abusoglu, Duygu Eryavuz Onmaz, Gulsum Abusoglu, Fatma Humeyra Yerlikaya, Ali Unlu

**Affiliations:** a Department of Biochemistry, Selcuk University Faculty of Medicine, Konya, Turkey; b Department of Medical Laboratory Techniques, Selcuk University Vocational School of Health, Konya, Turkey

**Keywords:** Tryptophan, Kynurenine pathway, Mass spectrometry, Validation

## Abstract

**Objectives:**

Recent studies have shown that derangements in kynurenine pathway metabolite levels are associated with various pathologies such as neurodegenerative diseases, schizophrenia, depression, bipolar disorder, rheumatoid arthritis, and cancer. Therefore, reliable, accurate, fast, and multiplex measurement methods for kynurenines have become increasingly important. This study aimed to validate a new mass spectrometric method for analyzing tryptophan metabolites.

**Methods:**

A tandem mass spectrometric method, including protein precipitation and evaporation steps, was developed to measure serum levels of tryptophan, kynurenine, kynurenic acid, 3-hydroxykynurenine, and 3-hydroxyanthranilic acid. Samples were separated using a Phenomenex Luna C18 reversed-phase column. The kynurenine pathway metabolites were detected by tandem mass spectrometry. The developed method was validated according to Clinical & Laboratory Standards Institute (CLSI) guidelines and applied to hemodialysis samples.

**Results:**

The developed method was linear at the concentrations of 48.8 – 25,000, 0.98 – 500, 1.2–5000, 1.2–5000, and 0.98–250 ng/mL for tryptophan, kynurenic acid, kynurenine, 3-hydroxyanthranilic acid, and 3-hydroxykynurenine, respectively. The imprecisions were less than 12 %. The median serum concentrations of tryptophan, kynurenine, kynurenic acid, 3-hydroxykynurenine, and 3-hydroxyanthranilic acid were 10530, 1100, 218, 17.6, and 25.4 ng/mL in pre-dialysis blood samples, respectively. They were 4560, 664, 135, 7.4, and 12.8 ng/mL in post-dialysis blood samples, respectively.

**Conclusions:**

A fast, simple, cost-effective, accurate, robust, and validated tandem mass spectrometric method was developed, and the method was successfully used for the quantitation of kynurenine pathway metabolite concentrations in hemodialysis patients.

## Highlights

• A multiplex LC-MS/MS method was developed for the measurement of kynurenines.
• Inta- and inter-assay imprecisions were less than 12% for all metabolites.
• Total run time was 5 min.
• The recoveries ranged between 94% and 105% and matrix effects were less than 6.7%.
• The method has simple pretreatment, and extraction recovery and sensitivity within acceptable ranges.

## Introduction

Tryptophan and its biological molecules have essential roles in metabolism. It is metabolized by four major biological pathways (serotonin, indole-3-pyruvate, kynurenine, and tryptamine). However, most of the tryptophan in the body is converted to kynurenine [1]. Tryptophan is converted to kynurenine by two enzymes (tryptophan 2,3-dioxygenase and indolamine 2,3-dioxygenase), followed by serial enzymatic reactions via riboflavin and pyridoxine as cofactors into kynurenic acid, anthranilic acid and their 3-hydroxy forms [2]. In addition to its structural participation in protein synthesis and being an essential amino acid, it has recently been popular for its role in psychiatric diseases [3]. Recent studies have revealed the importance of the kynurenine metabolic pathway in forming cancer, neurological and autoimmune diseases [4]. Many studies in the literature measure tryptophan and its metabolites using UV, fluorescence, electrochemical, and mass spectrometric methods [5]. High-performance liquid chromatography (HPLC) methods using UV detectors are among the preferred methods for clinical laboratories. However, it is a common problem that molecules with similar structures, mainly endogenous in the body, cause interference in analysis. Extended analysis time in UV detection is another significant problem [6]. The electrospray ionization technique (ESI) paired with liquid chromatography-mass spectrometry (LC-MS/MS) is a very sensitive and specific method that allows for multiplex analysis on many different sample types [7]. However, the tandem mass spectrometric methods reported to date are disadvantaged due to their low sensitivity, accuracy, extraction recovery and precision, and limited number of metabolites to be measured. In addition, they have time-consuming, costly pretreatment steps, extended analysis time, and require large amounts of samples [[8], [9], [10], [11], [12]]. The kynurenine pathway has been demonstrated to be involved in many diseases and disorders, including Alzheimer's disease, amyotrophic lateral sclerosis, Huntington's disease, AIDS dementia complex, malaria, cancer, depression, and schizophrenia [13]. Considering the comprehensive biological and pathological mechanisms of these metabolites, there is a need for reliable methods that allow for the measurement of these metabolites to be accurate, sensitive, and multiplex. Furthermore, it is possible to determine the levels of molecules in different metabolic pathways of the metabolism simultaneously with the sequential mass spectrometric method. Obtaining the levels of compounds in this pathway in a single chromatogram might give a broader overview of disease pathology [[14], [15]]. Therefore, this study aimed to develop and validate a multiplex, sensitive, cost-effective, practical, accurate, and reproducible tandem mass spectrometric method for the measurement of kynurenine pathway metabolites.

## Material and methods

### Chemicals

l-Tryptophan (CAS Number: 73–22-3), l-kynurenine (CAS Number 2922–83-0), kynurenic acid (CAS Number 492–27-3), 3-hydroxyanthranilic acid (CAS Number 548–93-6), 3-hydroxy-dl-kynurenine (CAS Number 484–78-6), HPLC grade water (CAS Number: 7732–18-5), acetonitrile (CAS Number 75–05-8), formic acid (CAS Number: 64–18-6) and donepezil hydrochloride (CAS Number 120011–70-3) were obtained from Sigma Aldrich.

### LC gradient set up and MS methods

**Fig. 1:**
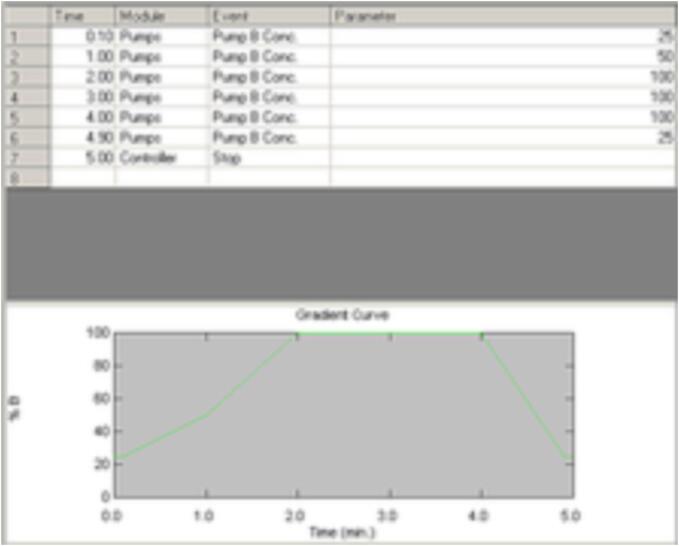
LC gradient for kynurenine pathway metabolites.

The separation of analytes was achieved using the C18 HPLC column (Phenomenex, 50 mm × 4.6 mm, part no: 00B-4041-E0), and the gradient elution of mobile phases A and B consisted of 100 % HPLC grade water with 0.1 % formic acid and 100 % acetonitrile with 0.1 % formic acid. The time-volume changes in the gradient program were as follows: 0–0.1 min., 25 % B; 1–2 min., 50 % B; 2–4 min., 100 % B; 4–5 min., 25 % B. Initially, isocratic elution was attempted. However, gradient elution was preferred as the metabolites could not be fully separated due to their physicochemical similarities. Different flow rates and run times from 1 min to 30 min were tried. The best peak shape and separation were achieved at a 0.8 mL / min flow rate and a run time of 5.0 min. The Shimadzu HPLC (Kyoto, Japan) system, coupled with a mass spectrometer, had a pump unit (LC-20 AD), an autosampler (SIL-20 AC HT), and an online degasser (DGU-20A3) unit. LC gradient for kynurenine pathway metabolites were shown in Fig. 1.

Kynurenine pathway metabolites were detected using an API 3200 (Applied Biosystems / MDS Sciex) tandem mass spectrometer set to ESI in positive multiple reaction monitoring (MRM) mode. The metabolites were identified by infusing pure standards. The Q1–Q3 ion transitions for tryptophan, kynurenic acid, kynurenine, 3-hydroxyanthranilic acid, 3-hydroxykynurenine and donepezil were 205.2/146.2, 190.2/144.0, 209.1/94.1, 154.0/136.0, 225.1/110.0, and 380.2/91.1, respectively. The main method optimization parameters for the mass spectrometer were set as follows: ionspray voltage of 5000 V; ion source temperature of 350 °C; gas1 and gas2 at 50 psi; curtain gas at 20 psi; and collision gas at 6 psi. Declustering entrance, collision cell exit potential, collision energy, entrance potential and dwell time for each analyte are shown in Table 1. Data analysis was processed by SCIEX Analyst® 1.6.2 Software, and analyte concentrations were calculated using the internal standard method.

**Table 1:** Tandem mass spectrometric method optimization parameters for kynurenine pathway metabolites.

**Analytes**	**Q1 (*m*/*z*)**	**Q3 (*m*/*z*)**	**Time(msec)**	**DP**	**EP**	**CE**	**CXP**
Tryptophan	205.2	146.2	400	39	10	25	3
Kynurenic acid	190.2	144.0	200	70	7.5	20	3
Kynurenine	209.1	94.1	400	41	10	26	3
3-hydroxyanthranilic acid	154.0	136.0	400	30	7.5	10	3
3-hydroxykynurenine	225.1	110	400	41.4	10	22.9	3
Donepezil	380.2	91.1	400	80	10	24	6

### Sample preparation

Serum metabolite concentrations were quantified using a modified method [16]. Briefly, 300 µL of the sample, 100 µL of donepezil (internal standard, 100 ng / ml), and 1000 µL of acetonitrile with 0.1 % (v/v%) formic acid were vortexed for 30 s. The mixture was then centrifuged at 2000 × *g* for 10 min, and the supernatants were evaporated with nitrogen gas at 37 °C. The residues were dissolved with a 200 µL acetonitrile:water (25:75; v/v) solution, including 0.1 % formic acid, and 30 µL of the mixture was injected into the system.

### Method validation

The tandem mass spectrometric method was primarily validated according to the Clinical and Laboratory Standards Institute (CLSI) C62-A: Liquid Chromatography-Mass Spectrometry Methods protocol [17]. Method validation parameters, such as linearity, precision, stability, recovery, carry-over, matrix effect, selectivity, and specificity, have been determined.

### Preparation of standard and working solutions

The calibration standards and working solutions were prepared with the help of a surrogate matrix [18]. Since the kynurenine pathway metabolites are endogenous compounds, a surrogate matrix was adopted to calibrate and prepare working solutions [19]. Different surrogate matrices, such as phosphate-buffered saline (PBS) solution (0.01 M phosphate buffer, 0.0027 M potassium chloride, 0.137 M sodium chloride, pH 7.4 at 25 °C) including 1 % bovine serum albumin (BSA), water, acetonitrile, and methanol were evaluated as candidate surrogate matrices. The PBS solution containing 1 % BSA was preferred as the surrogate matrix for validation studies because it had equivalent protein content and ionic strength to human plasma [20]. All solutions used for calibration and validation were freshly prepared by spiking analytes into the PBS solution, including 1 % BSA, and stored at + 4 °C. Standards at three different concentration levels (low, medium and high) were prepared by spiking into the PBS solution including 1 % BSA for each metabolite to demonstrate the equivalence between the selected surrogate matrix (1 % BSA solution) and real matrix (human serum). Therefore, a blank matrix was prepared using serum samples from at least six different blood donors for each metabolite, and baseline metabolite concentrations were measured. The percent of equivalence was calculated with the following formula [21]:

$$Equivalency\;\left(\%\right)=\left(\frac{Response\;serum\;spike\;-Response\;serum\;blank}{Response\;surrogate\;spike}\right)\times 100$$

Response serum spike was the peak area of the analyte-spiked serum pool. Response serum blank was the peak area of the analyte in the serum pool, and the surrogate response spike was the peak area of the analyte-spiked surrogate matrix. All metabolites' equivalency (%) ranged from 90 to 104 %. Consequently, the PBS solution, including 1 % BSA and human serum, had a similar matrix effect. Stock solutions of tryptophan, kynurenic acid, kynurenine, 3-hydroxyanthranilic acid, and 3-hydroxykynurenine were prepared at final concentrations of 25, 10, 10, 5, and 4 mg/mL, respectively. The calibration standards were prepared at concentration ranges of 48.8–25,000, 0.98–500, 1.2–5000, 1.2–5000, and 0.98–250 ng/mL for tryptophan, kynurenic acid, kynurenine, 3-hydroxyanthranilic acid, and 3-hydroxykynurenine via serial dilution of the stock solutions with the surrogate matrix. A 100 ng/mL internal standard (donepezil) solution was prepared in methanol. Prepared solutions were stored at 2 to 8 °C until analysis.

### Statistical analysis

SPSS statistical software package version 21.0, EP Evaluator Release 8 version (Data Innovations, South Burlington, VT), and Excel (2010) were used for the statistical evaluation, and a p-value less than 0.05 was accepted as statistically significant.

## Results

### Linearity

The linearity study followed the CLSI EP06-A protocol [17]. Calibration standards for tryptophan, kynurenic acid, kynurenine, 3-hydroxyanthranilic acid, and 3-hydroxykynurenine were prepared as described above. Calibration plots were set up by plotting the analyte/internal standard peak area ratios against the nominal metabolite concentrations. The linearity results were assessed using linear regression analysis. The correlation coefficients of the tryptophan, kynurenic acid, kynurenine, 3-hydroxyanthranilic acid, and 3-hydroxykynurenine calibration curves were found to be 0.9994, 0.9996, 0.9984, 0.9968 and 0.9976, respectively. The developed method was found to be linear across the entire range of the prepared calibration standards, respectively.

### LOD (Limit of detection) and LLOQ (Lower limit of quantitation)

There are various approaches to determining the LLOQ value. The first method is based on determining the lowest analyte concentration that can be quantified with satisfactory precision and accuracy. According to FDA guidelines, data from at least five replicates of the analyte-spiked sample in at least three independent runs should be used. Calculated imprecision values should be ≤ ±20 %, and a deviation in accuracy should be ≤ ±20 % [22]. Therefore, 20 samples were studied for five consecutive days to determine the LLOQ values, and intra- and inter-assay imprecisions and accuracy values were calculated. The accuracy study was performed by analyzing 20 replicates of a pre-treated low-level analyte-spiked PBS solution containing 1 % BSA, and the bias % values were calculated using the following formula: Bias%=.$\left(\frac{measured\;value\;-\;expected\;value}{expected\;value\;}\right)\times 100$

LLOQ values were determined as 48.8, 1.96, 2.4, 2.4, and 1.96 ng/mL for tryptophan, kynurenic acid, kynurenine, 3-hydroxyanthranilic acid, and 3-hydroxykynurenine, respectively. The intra- and inter-day imprecision values ranged from 7.12 % to 11.71 %, and accuracy values ranged from 88 % to 112 % for all analytes at LLOQ concentrations. The limits of blank (LOB) concentrations were determined after 20 replicates of analyte-free PBS solution containing 1 % BSA were analyzed. LOD values were determined after the analysis of 20 replicates of the low level analyte spiked PBS solution containing 1 % BSA. The following formulas were used to calculate LOB and LOD values: LOB = meanblank + 1.65SD_blank_ and LOD = LOB + 1.65SD_low concentration sample_ [23]. LOD values of tryptophan, kynurenic acid, kynurenine, 3-hydroxyanthranilic acid, and 3-hydroxykynurenine were 15.5, 0.90, 1.0, 1.0, 0.95 ng/mL, respectively.

### Intra- and inter-assay precision

This study was performed according to the CLSI EP05-A3 protocol [17]. Precision studies were carried out at low, medium, and high concentration levels determined for each metabolite throughout the calibration curve (Table 2). Inter-day imprecision was calculated based on 20 measurement results by analyzing four replicates per day for five consecutive days for each concentration level. Intra-day imprecision was calculated based on measuring 40 samples within a day. Then, imprecision values were determined using the following formula:

$$CV\%\;=\left(\frac{standard\;deviation}{mean\;}\right)\times 100$$

**Table 2:** Inta- and inter-assay precision study results of kynurenine pathway metabolites.

		**Intra-assay**				**Inter-assay**		
	Concentrations (ng/mL)	Mean	SD	CV%		Mean	SD	CV%
Tryptophan	6250	6300.3	274.4	3.34		6271	220.9	3.52
	781	781.1	32.1	4.10		783.5	38.1	4.87
	97.5	96.24	4.1	4.22		95.31	4.9	5.23
								
Kynurenic acid	250	243.5	9.2	3.77		244.8	10.5	4.28
	62.5	62.8	2.5	3.96		63.1	2.8	4.38
	15.6	15.67	0.76	4.85		14.69	0.74	5.06
Kynurenine	5000	5056.1	240.1	4.75		5073.6	232.3	4.58
	625	625.9	31.9	5.11		630.1	29.1	4.62
	78	79.1	3.7	5.15		80.21	4.1	5.68
								
3-hydroxyanthranilic acid	5000	5091.3	301.9	5.93		5054.2	266.3	5.27
	625	626.5	38.3	6.12		628.1	33.7	5.38
	78	76.4	4.5	6.66		77.0	4.2	6.72
								
3-hydroxykynurenine	250	255.6	8.9	3.48		257.1	7.8	3.10
	62.5	63.4	2.5	3.96		64.5	2.4	3.68
	15.6	15.8	0.64	4.05		15.8	0.66	4.20

The precision study results are presented in Table 2.

### Stability

The stability study followed (Table 3) the CLSI EP25-A protocol [17]. The effects of the freeze–thaw process and long-term storage (at −20 °C for 45 days) were evaluated. A long-term stability study was performed by analyzing the metabolite concentrations in the blank matrix obtained from 30 different serum samples on the collection day and then measuring the metabolite concentrations after storage at −20 °C on the 15th, 30th, and 45th days. The freeze–thaw stability was evaluated during 4 freeze–thaw cycles following the analyses of analyte concentrations on the collection day. The bias% values were calculated compared to the measured metabolite levels on the collection day (expected value) via the following formula:

$$Bias\%=\left(\frac{measured\;value\;-\;expected\;value}{expected\;value\;}\right)\times 100$$

**Table 3:** Stability study bias% results of kynurenine pathway metabolites.

		**Frozen (-20 ^◦^C) for 45 day**				**Freeze-thaw stability**		
	Concentrations (ng/mL)	15.Day (%)	30.Day (%)	45.Day (%)		2. (%)	3. (%)	4. (%)
Tryptophan	6250	2.63	5.89	8.7		−3.5	−6.24	−10.5
	97.5	3.89	5.25	7.6		−2.33	−5.65	−11.6
Kynurenic acid	250	3.60	4.95	10.9		−1.8	−4.7	−10.5
	15.6	3.21	5.41	12.6		−2.9	−5.4	−12.7
Kynurenine	5000	3.40	5.20	11.8		1.6	6.7	11.9
	78	3.05	4.75	9.7		2.3	6.2	12.4
3-Hydroxyanthranilic acid	5000	−2.59	−5.86	−12.3		−1.5	−4.68	−10.1
	78	−2.73	−6.12	−10.2		2.6	5.1	−11.2
3-Hydroxykynurenine	250	−2.62	−5.4	−11.4		−2.2	−5.95	−12.8
	15.6	−1.85	−3.65	−9.6		−2.0	−4.65	−11.5

All metabolites were found to be stable during 45 days and four freeze–thaw processes.

### Recovery and matrix effects

The recovery study followed the CLSI EP34 protocol [17]. The recovery study used three different concentration levels of each analyte (Table 4). The results were calculated using the following formula: Recovery%=[(*C*2 − C0)/C1] × 100.

**Table 4:** The results of the recovery and matrix effect studies for kynurenine pathway metabolites.

**Analytes**	**Concentrations (ng/ml)**	**Recovery%**	**Matrix effect%**
Tryptophan	6250	102.6	3.7
	781	98.7	4.2
	97.5	99.5	5.8
Kynurenic acid	250	94.3	3.4
	62.5	95.9	4.6
	15.6	97.7	4.9
Kynurenine	5000	102.3	3.2
	625	101.8	5.2
	78	104.8	5.1
3-hydroxyanthranilic acid	5000	104.2	6.6
	625	97.4	5.9
	78	100.8	6.3
3-hydroxykynurenine	250	101.5	4.5
	62.5	98.6	5.2
	15.6	97.5	6.3

C2 was the measured analyte concentration after spiking, C0 was the initial analyte concentration of the solution, and C1 was the added known concentration of standard.

The matrix effect is generally evaluated with one of two standard methods: post-column infusion or post-extraction spiking. In this study, the second method was preferred, as reported by Chambers et al. [24]. In this context, the low, medium, and high-level analyte response in neat solutions (such as water, mobile phase mixture, acetonitrile, and methanol) was compared with the analyte response in the spiked matrix at equal levels after pretreatment. Firstly, three different concentration levels were obtained by spiking low, medium, and high (Table 4) level analytes into a mixture of water and methanol (50:50, v/v%) to prepare the neat solution. Secondly, a surrogate matrix PBS solution containing 1 % BSA was pretreated, and after the residues were dissolved with a mobile phase mixture, equal concentration levels to the neat solution were prepared. The matrix effect values were calculated using the following formula: ME% = (Mean post-extracted peak area / Mean un-extracted peak area) × 100.

The results of the recovery and matrix effect studies are shown in Table 4.

Results of the matrix effect study. Matrix effects were less than 6.7 % for all metabolites.

### Carry-over

The carry-over study analyzed low- and high-level samples in the order stated in CLSI EP10-A3 [17]. Low concentration levels were fixed in PBS, including BSA for tryptophan, kynurenic acid, kynurenine, 3-hydroxyanthranilic acid, and 3-hydroxykynurenine at 97.5, 15.6, 78, 78, and 15.6 ng/mL, respectively. High concentration levels were fixed in PBS solution including BSA for tryptophan, kynurenic acid, kynurenine, 3-hydroxyanthranilic acid, and 3-hydroxykynurenine at 6250, 250, 5000, 5000, and 250 ng/mL respectively. After performing the pretreatment steps, high and low concentration levels were analyzed in a specified order. The carry-over study was conducted individually for each analyte. The orders of samples were expressed below.

L1-L2-L3-H1-H2-L4-H3-H4-L5-L6-L7-L8-H5-H6-L9-H7-H8-L10-H9-H10-L11.

The calculated results from the tandem mass analysis were recorded onto the EP Evaluator Release 8 program (Data Innovations, South Burlington, VT) and carry-over results were determined for each analyte. The EP Evaluator program determined the acceptability criteria based on CLSI protocol EP10-A3 guidelines.

The carry-over values are shown in Table 5.

**Table 5:** The carry-over study results of kynurenine pathway metabolites.

**Analytes**		**Low-low results (ng/ml)**		**High-low results (ng/ml)**			**Carry-over (ng/ml)**
		Mean	SD	Mean	SD		
Tryptophan		98.7	4.38	96.42	5.43		−2.28
Kynurenic acid		15.54	0.59	15.93	0.93		0.39
Kynurenine		78.18	2.67	80.38	1.24		2.20
3-hydroxyanthranilic acid		78.63	2.93	82.14	1.54		3.51
3-hydroxykynurenine		15.64	0.66	16.21	0.34		0.57

### Application of the method for patient samples

**Fig. 2:**
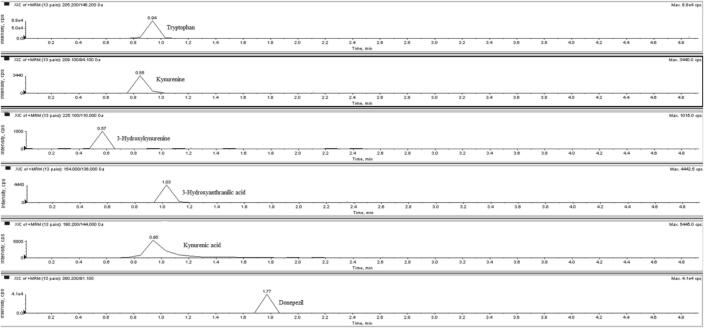
The chromatograms of kynurenine pathway metabolites obtained from a patient serum sample before dialysis.

**Fig. 3:**
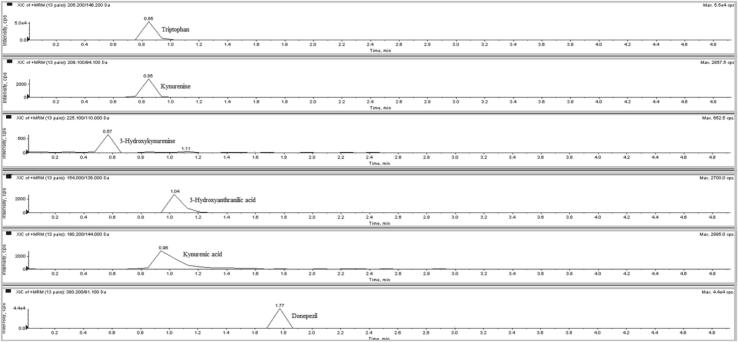
The chromatograms of kynurenine pathway metabolites obtained from a patient serum sample after dialysis.

The validated method was applied to clinical samples obtained from patients with chronic kidney disease (CKD) who had been undergoing hemodialysis for at least three months in the dialysis unit of Selcuk University Faculty of Medicine Hospital. Two milliliters of blood were drawn into collection tubes, and then centrifuged at 2000 *g* for 10 min. Patient samples were kept at −80 °C until the day of analysis. This study was approved by Selcuk University's local ethics committee (Number: 2020/582, Date: 30/12/2020). The chromatograms of kynurenine pathway metabolites obtained from a patient serum sample before and after dialysis are shown in Fig. 2, Fig. 3, respectively.

The kynurenine pathway metabolite levels and routine laboratory parameters measured in post- and pre-dialysis serum samples are presented in Table 6.

**Table 6:** Pre- and post- hemodialysis levels of kynurenine pathway metabolites, creatinine, urea, potassium and eGFR levels in patients with chronic renal failure.

Metabolite	Pre-hemodialysis	Post-hemodialysis	P
eGFR (ml/min/1.73 m2)	7.3 (3.9–15.9)	29.7 (11.7–63.0)	**<0.001**
Creatinine (mg/dL)	7.0 (3.7–12.8)	2.3 (1.2–5.6)	**<0.001**
Urea (mg/dL)	98 (54–165)	24 (11–52)	**<0.001**
Potassium (mmol/L)	4.9 (4–6.6)	3.6 (2.7–4.9)	**<0.001**
Tryptophan (ng/mL)	10,530 (3522–20640)	4560 (1536–12360)	**<0.001**
Kynurenine (ng/mL)	1100 (400–1590)	664 (356–1020)	**<0.001**
Kynurenic acid (ng/mL)	218 (100–551)	135 (46.1–340)	**<0.001**
3-hydroxyanthranilic acid (ng/mL)	17.6 (5.7–137)	7.4 (1.3–37)	**<0.001**
3-hydroxykynurenine (ng/mL)	25.4 (7.2–90.1)	12.8 (2.2–47.5)	**<0.001**

## Discussion

Tryptophan is an essential amino acid that is important for living organisms. Less than 1 % of the available tryptophan is used for protein synthesis [25]. Tryptophan's main metabolic route is the kynurenine pathway, through which approximately 90–95 % of tryptophan degrades into nicotinamide adenine dinucleotide (NAD + ). The degradation of tryptophan by the kynurenine pathway causes the generation of various intermediates with neuroactive, immunoregulatory, oxidant, or antioxidant effects, such as kynurenine, kynurenic acid, 3-hydroxykynurenine, and 3-hydroxyanthranilic acid before NAD^+^ [26]. Since kynurenine pathway metabolites have physiologically important and sometimes opposing effects, their levels are tightly regulated. Imbalances in the kynurenine pathway metabolite levels can cause various pathologies, such as Alzheimer's, Huntington's, Parkinson's, schizophrenia, depression, bipolar disorder, rheumatoid arthritis, and cancer [[27], [28], [29], [30]]. Therefore, the importance of reliable, accurate, rapid, and multiplex measurement methods for kynurenines has increased.

However, quantifying tryptophan and metabolite levels is challenging due to their low stability, low blood concentrations, and presence of interferences in biological matrices. Since each of the kynurenine pathway metabolites has different physiologically characteristic functions, it is vital that most of the metabolites are able to be quantified by a simultaneous measurement method. However, an important aspect of the problem is that the physiological concentrations of metabolites vary over a wide range; for example, tryptophan is found in micromolar concentrations, while other kynurenine pathway metabolites are found in nanomolar concentrations. Another critical problem is the need for selective and specific methods because these metabolites have similar physical properties and structures [[6], [31]]. While HPLC-based techniques such as HPLC-UV [32], HPLC-FD [33], and HPLC-ECD [[34], [35]] have been commonly used in the measurement of kynurenine pathway metabolite levels, these methods have some essential disadvantages. For example, HPLC-UV methods are disadvantageous due to their low sensitivity, specificity, and selectivity. The risk of interference from matrix components is higher in HPLC-UV methods. The relatively long analysis time is another problem with these methods. Although HPLC-FD methods have sufficient sensitivity, they have disadvantages due to complex and time-consuming pretreatment steps that require derivatization. Although HPLC-ECD methods also have high sensitivity, the main disadvantage is the low reproducibility caused by electrode clogging and loss of selectivity [[6], [31]]. However, LC-MS/MS allows for the simultaneous measurement of kynurenine pathway metabolites in a single experiment. This method has advantages such as good separation ability, high selectivity, specificity, and sensitivity; it requires a small amount of sample; and has a short run time and practical pretreatment steps. Various LC-MS/MS methods are reported for measuring various kynurenine pathway metabolites [[2], [7], [8], [9], [10], [11], [12]].

Möller et al. revealed a tandem mass method for simultaneously measuring six different kynurenine pathway metabolites, including tryptophan, kynurenine, kynurenic acid, anthranilic acid, 3-hydroxyanthranilic acid, and quinolinic acid. Intra- and inter-assay imprecision values of the method ranged from 1.0 % to 9.9 %, and accuracy values ranged from 75 % to 107 %. The mean recovery values were between 96 % and 101 %, and the LLOQ values of tryptophan, kynurenine, kynurenic acid, anthranilic acid, 3-hydroxyanthranilic acid, and quinolinic acid were 0.0352 μM, 0.035 μM, 0.035 μM, 0.047 μM, 0.084 μM, and 0.096 μM, respectively. The method had advantages such as sufficient sensitivity, good recovery, and accuracy; however, its disadvantages were the required high amount of sample (800 µL), time-consuming pretreatment steps including solid phase extraction (SPE), and long run time (12 min) [8]. Fuertig et al. reported a tandem mass method based on protein precipitation for the multiplex analysis of tryptophan, kynurenine, 3-hydroxykynurenine, 3-hydroxyanthranilic acid, quinolinic acid, picolinic acid, kynurenic acid, xanthurenic acid, serotonin, dopamine, and neopterin levels. Intra- and inter-assay imprecision of this method for all analytes ranged from 1 % to 23 %, while accuracy values ranged from 42 % to 116 %. The LOQ values ranged between 0.25 and 2.5 nM. The significant advantages of the method were excellent accuracy, precision (except for 3-hydroxyantranilic acid), short run time (5.5 min), and sufficient sensitivity; however, it had time-consuming processing steps requiring a 30 min incubation at −20 °C and evaporation of samples [9]. Huang et al. reported an LC-MS/MS method based on protein precipitation with trifluoroacetic acid to quantify plasma tryptophan and kynurenine levels. The intra- and inter-assay imprecisions of the method were less than 7.1 %, and the accuracy values were between 89 % and 108 %. The LOQ values of tryptophan and kynurenine were 1250 ng/mL and 62.5 ng/mL, respectively. The analysis time of each sample was 4.5 min [10]. Similarly, Miller et al. reported a tandem mass spectrometric method for quantifying tryptophan and kynurenine levels based on precipitation with trifluoroacetic acid. The extraction recovery of the method was between 89 % and 95 %, and the intra- and inter-assay imprecision values were less than 7 %. The run time was approximately 4.1 min. This method had a short analysis time, simple sample preparation procedures, good precision, extraction recovery, and accuracy; however, its low sensitivity and lack of other metabolites were limitations [11]. Hu et al. reported a tandem mass spectrometric method including protein precipitation followed by evaporation steps. This method's intra- and inter-assay imprecision values were less than 11 %, and the accuracy values were between 88 % and 107 %. The mean recovery values ranged from 92 % to 101 %. The LOQ values were 1000, 100, and 1 ng/mL for tryptophan, kynurenine, and kynurenic acid, respectively. The chromatographic run time was 4.2 min. This method had good extraction recovery, precision, simple pretreatment steps, and moderate sensitivity [12]. Tong et al. reported an LC-MS/MS method for simultaneously determining tryptophan, kynurenine, kynurenic acid, xanthurenic acid, and 5-hydroxytryptamine in human plasma. The mean recovery values ranged between 82 % and 107 %, and intra- and inter-assay imprecision values were less than 13.2 %. Simple pretreatment steps including protein precipitation and evaporation; short run time (3.5 min); good precision; and extraction recovery are the main advantages of this method [16]. The number of methods for quantifying different kynurenine pathway metabolites may still increase. We have developed a tandem mass spectrometric method for the multiplex analysis of serum tryptophan, kynurenine, kynurenic acid, 3-hydroxykynurenine, and 3-hydroxyanthranilic acid levels. The pretreatment steps were based on protein precipitation and evaporation. LLOQ values were determined as 48.8, 1.96, 2.4, 2.4, 1.96 ng/mL for tryptophan, kynurenic acid, kynurenine, 3-hydroxyanthranilic acid, and 3-hydroxykynurenine respectively. The intra- and inter-day imprecisions were less than 12 %, and the accuracy values ranged between 88 % and 112 %. The recovery values ranged between 94 % and 105 %, and the matrix effect values were less than 6.7 % for all analytes. This method was advantageous regarding sufficient sensitivity, simple sample processing, short run time (5 min), good extraction recovery, accuracy, and precision.

The second important aspect of our study was the clinical application of a validated method for measuring kynurenine pathway metabolite levels in pre- and post-dialysis serum samples from patients with CKD who had undergone hemodialysis. There are few studies investigating kynurenine pathway metabolite levels in patients with CKD. Debnath et al. reported mean serum tryptophan, kynurenine, 3-hydroxykynurenine, kynurenic acid, and quinolinic acid levels in patients with CKD secondary to type 2 diabetes as 10,030, 790.4, 2,336, 26.9, and 611.2 ng/mL, respectively [36]. Konje et al. reported that the mean serum tryptophan, kynurenine, and 3-hydroxyanthranilic acid levels of patients with stage 1 and stage 2 CKD were 14,178, 811.2, and 8.72 ng/mL, and the median kynurenic acid and quinolinic acid levels were 0.051 and 35 ng/mL. The levels of these metabolites for CKD stage 3 patients were 14,565, 1,060, 8.72, 0.18, and 38 ng/mL, while they were 13,301, 1,165, 13.38, 0.36, and 38.4 ng/mL for CKD stage 4 and 5 patients, respectively [37]. Pawlak et al. reported the median levels of serum kynurenine, 3-hydroxykynurenine, kynurenic acid, and quinolinic acid as 638.5, 45.5, 3.9, and 25.1 ng/mL in patients with hemodialysis [38]. Malhotra et al. reported serum tryptophan and kynurenine levels of hemodialysis patients as 10,689 and 665.6 ng/mL [39]. Yilmaz et al. found the mean serum tryptophan and kynurenine levels as 5295 and 844.5 ng/mL in hemodialysis patients, respectively [40]. The median serum values of tryptophan, kynurenine, kynurenic acid, 3-hydroxykynurenine, and 3-hydroxyanthranilic acid levels were determined as 10,530, 1,100, 218, 17.6, and 25.4 ng/mL in pre-dialysis blood samples respectively in our study. The levels of these metabolites in post-dialysis blood samples were 4,560, 664, 135, 7.4, and 12.8 ng/mL respectively. Our results were consistent with previous studies; however our findings showed that serum metabolite levels decreased approximately twofold post-dialysis compared to pre-dialysis samples.

## Conclusions

A validated LC-MS/MS method was developed to quantify concentrations of metabolites in the kynurenine pathway. It was then applied to clinical samples obtained from hemodialysis patients. The primary advantages of the method were its short run time, simple pretreatment steps, cost-effectiveness, good extraction recovery, high precision, sufficient sensitivity, and ability to provide multiplex analysis with enough selectivity. The metabolite levels measured in hemodialysis patients were consistent with those of previous studies, meaning that the developed method can be reliably used in clinical samples.

## CRediT authorship contribution statement

**Sedat Abusoglu:** Conceptualization, Writing – original draft, Visualization. **Duygu Eryavuz Onmaz:** Conceptualization, Writing – review & editing. **Gulsum Abusoglu:** Investigation, Methodology. **Fatma Humeyra Yerlikaya:** Writing – original draft, Visualization. **Ali Unlu:** Supervision, Project administration.

## Declaration of Competing Interest

The authors declare that they have no known competing financial interests or personal relationships that could have appeared to influence the work reported in this paper.
